# Comparative, prospective, and randomized study between urotherapy and the pharmacological treatment of children with urinary incontinence

**DOI:** 10.1590/S1679-45082013000200012

**Published:** 2013

**Authors:** Renata Martins Campos, Antonio Gugliotta, Osamu Ikari, Maria Carolina Perissinoto, Adélia Correia Lúcio, Ricardo Miyaoka, Carlos Arturo Levi D'Ancona

**Affiliations:** 1 Universidade Estadual de Campinas, Campinas, SP, Brazil

**Keywords:** Enuresis, Urinary incontinence, Physical therapy modalities

## Abstract

**Objective:**

To verify and compare the results of behavioral modification plus pelvic floor muscle training and behavioral modifications plus oxybutynin chloride in children with nonmonosymptomatic enuresis.

**Methods:**

A total of 47 children were randomized using opaque and sealed envelopes sequentially numbered. Group I was composed of 21 children who underwent antimuscarinic treatment (oxybutynin), and Group II was composed of 26 patients who underwent pelvic floor muscle training. Both groups were instructed as to behavioral modifications.

**Results:**

The voiding diary results were compared each month between Groups I and II. In the first month of treatment, children in Group I presented 12.2 dry nights, 13.4 in the second month, and 15.9 in the last month. In Group II, the results were: 14.9 dry nights in the first month, 20.8 dry nights in the second and 24.0 dry nights in the last month. There was a significant difference between the groups in second and third months.

**Conclusion:**

Pelvic floor exercises associated with behavioral changes were more effective than pharmacological treatment in children with urinary incontinence.

## RESUMO

**Objetivo:**

Verificar e comparar os resultados da modificação comportamental associado ao treinamento dos músculos do assoalho pélvico e modificação comportamental associado ao uso de cloridrato de oxibutinina em crianças com enurese não monossintomática.

**Métodos:**

Foram randomizadas 47 crianças por meio de envelopes opacos e selados com numeração sequencial. O Grupo I foi composto por 21 crianças que receberam tratamento com antimuscarínico (oxibutinina) e o Grupo II por 26 pacientes que receberam treinamento dos músculos do assoalho pélvico. Ambos os grupos foram instruídos em relação à modificação comportamental.

**Resultados:**

Os resultados do diário miccional foram comparados cada mês entre os Grupos I e II. No primeiro mês de tratamento, as crianças do Grupo I apresentaram 12,2 noites secas, 13,4 no segundo mês e 15,9 no último mês. No Grupo II, os resultados foram: 14,9 noites secas no primeiro mês, 20,8 no segundo mês e 24,0 no último mês. Houve diferença significativa entre os grupos no segundo e no terceiro mês.

**Conclusão:**

Os exercícios do assoalho pélvico associados a mudança comportamental foram mais efetivos do que o tratamento farmacológico em crianças com incontinência urinária.

## INTRODUCTION

According to the International Children's Continence Society (ICCS), nocturnal incontinence, or enuresis, denotes urinary incontinence (UI) during sleep. Incontinence during the day is defined as daytime incontinence^(1)^. The new subdivision recommended by ICCS is enuresis in children without any other low urinary tract symptoms (LUTS); without a history of bladder dysfunction, it is defined as monosymptomatic enuresis. Other cases with enuresis and any other LUTS are classified as nonmonosymptomatic enuresis^(1)^.

UI due to non-neurogenic bladder dysfunction is a frequent bother in children. The prevalence of monosymptomatic enuresis in children between 6 and 12 years ranges from 0.2 to 9.0%, and 1.5 to 2.8% for nonmonosymptomatic enuresis^(2)^.

The etiology of enuresis is multifactorial and hypothesized to be related to problems with arousal, small bladder capacity, and large overnight urine production.

Daytime incontinence is associated with various comorbid conditions such as urinary tract infection, vesicoureteral reflux, constipation, and behavioral troubles^(3)^.

Children with nonmonosymtomatic enuresis treated with oxybutynin alone had a 54% success rate^(4)^. Pelvic floor exercise were introduced to pediatric urology by Wennergren and Oberg, with the aim of increasing children's awareness of the pelvic floor musculature and teaching them how to contract and relax these musculature at will^(5)^.

Urotherapy is a new terminology that includes information on and demystification of the voiding function and dysfunction, instruction on voiding habits, lifestyle advice regarding fluid intake, prevention of constipation, recording of symptoms and voiding habits in bladder diaries, and support via regular follow-up by a caregiver. Specific interventions include various forms of pelvic floor training^(1)^. Urotherapy is successful for the treatment of nonmonosymtomatic enuresis, achieving 42% of cases completely dry^(6)^. Resting pressure of the incontinent children was significantly improved by pelvic floor muscle exercise^(7)^.

## OBJECTIVE

To verify and compare the results of behavioral modification plus pelvic floor muscle training, and behavioral modifications plus oxybutynin chloride in children with nonmonosymptomatic enuresis.

## METHODS

The study was carried out at the Division of Urology at the *Universidade Estadual de Campinas* (UNICAMP) and it was approved by the Ethics in Research Committee under the number 555-2006. Parents signed the Informed Consent Form. This study comprised 47 children with nonmonosymptomatic enuresis and their mean age ranged from 5 to 10 years. Among them, 29 were girls. All children were evaluated by anamnesis, urinalysis, urine culture, and playful voiding diary. The children were randomized into two groups. The randomization was done using opaque and sealed envelopes sequentially numbered. Group I was composed of 21 children who received oxybutynin chloride treatment; Group II was composed of 26 patients who received pelvic floor muscle training. Both groups were instructed in behavioral modifications.

Inclusion criteria were nonmonosymptomatic enuresis, absence of prior treatment for UI, age between five and ten years old, and signed Inform Consent. The exclusion criteria were neurological disease, anatomical abnormalities, or urinary tract infection (UTI).

### Behavioral modification

#### Hygienic-dietetic

Counseling was used aiming to reeducate children's habits, involving orientation on ingestion of liquids and scheduled time to void with parents' help or the use of a timepiece. Recommendation on hydric ingestion and types of liquid preferred by the children, such as milk, soft drinks, juice, water, tea, and coffee, were evaluated. The period of greater ingestion of each liquid was planned. Parents were instructed to offer liquids that contained caffeine only in the morning and afternoon; soft drinks only after lunch. Only juice and water should be offered with no restriction, since several children did not have the correct ingestion of liquids for their age.

The use of bottles (of approximately 500mL) was also adapted for control of amount of ingested liquids per 24 hours, divided proportionally as 40 to 50% in the morning, 30 to 40% in the afternoon, and 10 to 20% in the evening.

#### Voiding position

**Figure 1:**
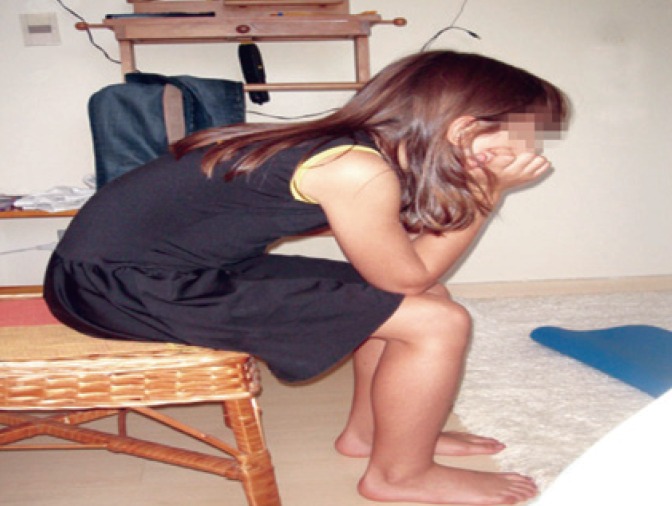
Position for voiding

For girls, postural adaptation involved relaxation of the pelvic floor muscles through toilet position while sitting on the toilet, with feet support on a surface or on the floor. The underwear was lowered down to the ankles, supporting the elbows on the knees and inclining their trunk to the front. In order to stimulate the children's patience, the children sang songs or counted numbers while waiting to void. Counseling for boys is the same as for girls, without sitting in the toilet (Figure 1).

#### Playful voiding diary

**Figure 2:**
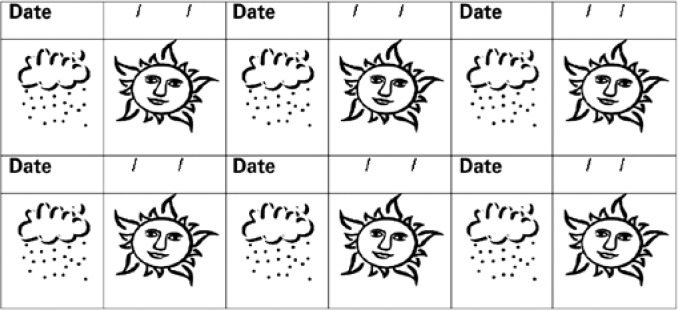
Ludic voiding diary

Children in both groups filled out a playful voiding diary. They were responsible for coloring the diary containing information about the night before. If they had a dry night, they colored the sun, and if they had a wet night, they colored the rain (Figure 2).

Urinary symptoms were evaluated by frequency, and day-time and night-time incontinence.

#### Group I

The oxybutynin chloride was used at the dose of 0.2mg/ kg, split into two times a day, during 3 months.

#### Group II

**Figure 3:**
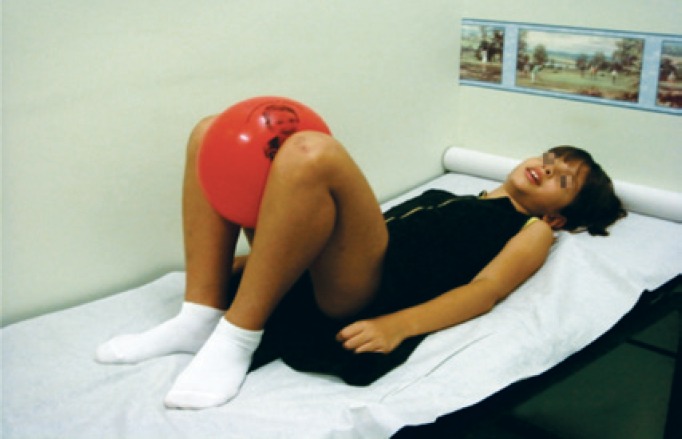
Pelvic floor muscles training

Pelvic floor muscle training rehabilitation programs are directed mainly at pelvic floor muscle recognition of contraction and relaxation these by squeezing a ball between the knees. There is concomitant work with the adductor muscles (Figure 3).

**Figure 4:**
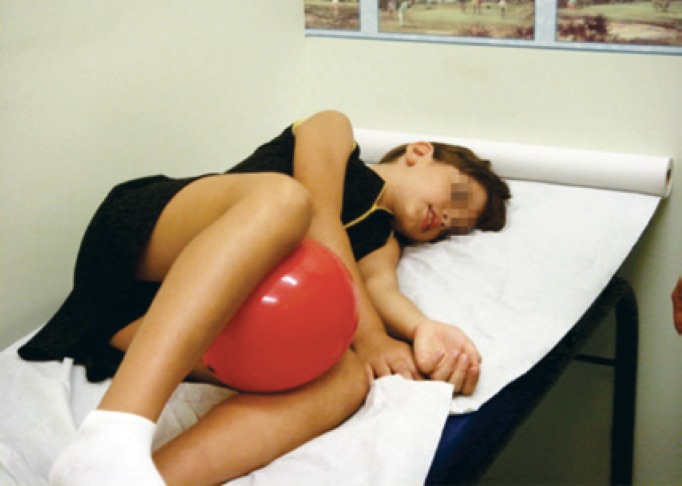
Abdominal muscles training

Lower abdominal (transversus and obliquous internus abdominis) and pelvic floor muscles act synergistically, and it is important that both are relaxed during voiding. Diaphragmatic breathing exercises were easy to learn and served to teach the children abdominal relaxation (Figure 4).

**Figure 5:**
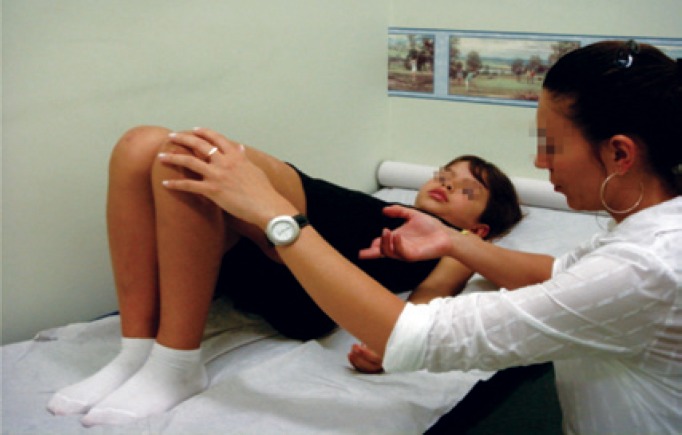
Gluteal muscles training

Two other muscles included in the program training are the gluteus muscles that are accessories (Figure 5).

Parent orientation on the exercise program allowed their direct participation in the treatment, which should be performed twice a week at home and once a week with the Physical Therapist. On the weekends no exercises were performed. All children were evaluated every month until the end of the study after 3 months.

### Statistical analysis

The χ^2^ or Fisher's exact test was used to compare proportions between the groups. The Mann-Whitney test was used to compare numerical variables. The variance analysis for repeated measures (ANOVA) was used to compare the number of dry nights recorded. Tukey's test was used each time to compare the groups. The variables were transformed into ranks due to the absence of normal distribution. The significance level adopted for the statistical tests was of 5% (p<0.05).

## RESULTS

Table 1 shows if the two groups were homogeneous regarding age, gender, and night-time continence. There was a higher incidence of girls in the groups studied: 29 (61.70%).

**Table 1:** Patient's data

	Group I (n=21)	Group II (n=26)	p value
Age (years)	9 (3-10)	8.5 (5-10)	0.876
Gender (girls)	13 (61.9%)	16 (61.54%)	0.626
Frequency	6 (3-12)	4.5 (2-20)	0.164
Nighttime incontinence	2 (1-4)	2 (1-7)	0.771
Daytime incontinence	3 (2-6)	3 (1-12)	0.759
Baseline days of continence	1 (0-2)	1 (0-3)	0.299

Mann-Whitney Test.

Median (minimum to maximum).

Thirty children drank liquids all day, without concerns about their UI. The analysis of beverage choice is shown on table 2. The volume of consumed liquid per day was 1.7±0.6L in Group I and 1.4±0.6L in Group II (p=0.055).

**Table 2:** Beverage choice of patients

	Group I n (%)	Group II n (%)	p value
Milk	13 (61.9)	20 (76.9)	0.263
Juice	12 (57.1)	11 (42.3)	0.312
Water	6 (28.6)	7 (26.9)	0.900
Tea	2 (9.52)	2 (7.69)	0.10
Soft drink	18 (85.7)	15 (57.7)	0.037

Pearson's χ^2^ test.

All children in Group I completed the treatment with no complaints about side effects and there were no dropouts. In Group II, three children interrupted the study due to difficulties in returning to the clinics.

According to the playful voiding diary, the improvement of continence during follow-up in Group I ranged from 11 (1 to 24) at the start of the study to 16 (0 to 27) days of continence per month at the last evaluation. In Group II, the results started at 15.5 (0 to 27) and progressed to 24.5 (6 to 30) days of continence per month. Comparing the two groups, the pelvic floor muscle training treatment showed significant improvement (p<0.001).

Furthermore, the playful voiding diary also reported the number of dry nights observed during 3 months of treatment by means of drawings in which the child colored in the weeks and the results were compared each month between Groups I and II. Children in Group I (oxybutynin) presented with 12.2 dry nights in the first month of treatment, 13.4 in the second month, and 15.9 in the last month. In Group II (urotherapy), the results were 14.9 dry nights in the first month, 20.8 dry nights in the second, and 24.0 dry nights in the last month. There was a significant difference between the groups in months 2 and 3.

In the late post-treatment after 4 months, the patients were contacted by phone and the parents informed their children's condition. They were asked whether the children were continent during the day and at night. In Group I, in which children were treated with oxybutynin and behavioral therapy, only 7 (31.83%) children were continent day and night. In Group II, 14 (58.33%) children who underwent pelvic floor muscle training and behavioral therapy were cured. The study showed a slightly significant tendency towards the children who were treated with urophysiotherapy – Group II (p=0.071).

## DISCUSSION

Lower urinary tract conditions are a common problem in children, with a prevalence rate of up to 15%^(8)^. From 7 to 10% of school-age children have recurrent urinary tract infections or UI based on non-neurogenic lower urinary tract dysfunction. UI prevalence differs among populations, and we believe that it is between 15 and 33% at the age of 5 years^(3)^.

Parents usually ignore daytime incontinence, since modern life and daily tasks lead to lack of participation of parents in family life. The detailed anamnesis reveals complaints about their constant bedwetting and wet linens, interruption of parents' sleep, and reduction of the child's self-esteem^(8)^.

According to Robson and Leung, daytime wetting is a common problem with various causes that can usually be identified through a carefully history, physical examination, and urinalysis^(9)^.

In this study, all children were instructed on behavioral therapy. Other authors confirm the importance of these recommendations showing reduced urinary losses when they were instructed to change their habits through reeducation about going to the bathroom every 1 or 2 hours^(10)^.

The toilet seating position with the feet supported is ideal for total relaxation of pelvic floor muscles for easier voiding. In another study, it was observed that the flow curve during voiding, after an adequate toilet posture in order to reach an optimal relaxation of the pelvic-floor, improved the flow. The individually adapted voiding allowed each child to deal consciously with the bladder and its function, and a number of simple rules for application at home increased the involvement and motivation of the child^(11)^.

The playful voiding diary attracted the interest of children, regarding the treatment method, and provided information about the frequency of UI^(11)^.

The evaluation concerning the types of consumed liquid and interval periods was an important point of this study, taking into account adequate hydration for children. Therefore, the parents were instructed as to behavioral change regarding the ingestion of liquids and voiding frequency. According to another study, the children ingested two glasses of liquid at each meal only during the day, showing flexibility regarding the types of liquid, such as tea, coffee, and soft drinks^(11)^.

Oxybutynin is an anticholinergic drug that has not been proven to be effective for treatment of nocturnal enuresis not accompanied by daytime symptoms, such as urgency. It can be added as a second-line drug and is effective for treating children with both daytime and nighttime wetting^(12)^.

The nonmonosymptomatic enuresis treatments included behavioral modification, biofeedback, antibiotics, anticholinergics, counseling, and neuromodulation. The antimuscarinics oxybutynin and tolterodine are, at present, the most commonly used drugs to treat incontinence. Common side effects with these agents (i.e., reduced saliva production and worsened constipation) can be severe and can cause up 10% of children using oxybutynin to discontinue treatment^(13)^.

Our study showed that the pelvic floor muscle exercise is an alternative treatment in these cases, demonstrating that pelvic floor work is not only useful for women and men, but also for children. It was applied in children due to its efficacy, since the voluntary contractions of pelvic floor muscles are reflexively followed by a relaxation of the detrusor muscle, inhibiting involuntary bladder contractions and suppressing the desire to urinate in incontinent children^(3)^.

Yamanishi et al. observe during biofeedback that patients in training were instructed to contract the anal sphincter without raising abdominal pressure to inhibit overactive bladder contractions^(14)^.

Sphincter relaxation was very important for effectiveness of the bladder contractions and its coordination in the voiding process. Due to this factor, this study adapted the voiding posture through adequate positioning on the toilet for all pelvic floor relaxation. In addition, through the ball exercises, it was possible to teach the children how to contract and relax their perineum, promoting voiding coordination. Austin and Coplen showed an increasing ability to voluntary contract the external striated sphincter over each year of life^(15)^.

**Figure 6:**
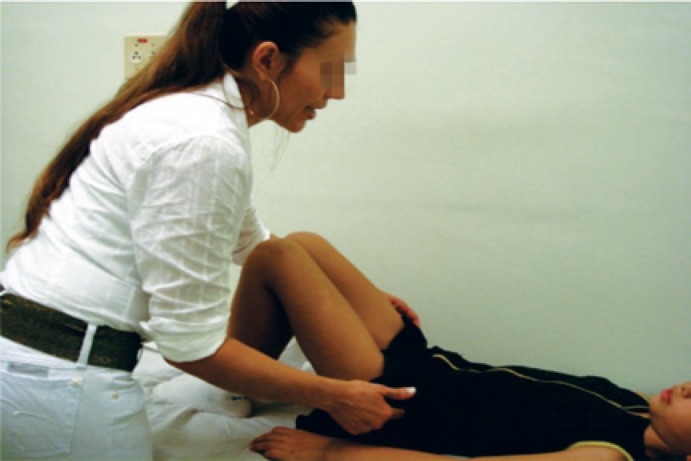
Pelvic and hip movements reeducation

Sapsford et al.^(16)^ confirmed that there was a synergic mechanical action with an increase in an intra-abdominal pressure, suggesting that the action of pelvic floor muscles occurred in a progression between both. The contribution of adductors and gluteus maximus was investigated, placing a surface electrode for observation of the interrelation with the pelvic floor muscles, and the contribution of these during the muscle contraction was registered. According to these techniques, the exercises with the children were performed separately, in accordance with different decubitus positions. The exercises were adapted to the groups according to age, to avoid exhaustion. It was also emphasized that the pelvic floor muscle exercises should be performed separately from the abdominal muscles and other hip muscles (Figure 6). We could observe that when performed together, the exercises increased the intra-abdominal pressure, overcharging the bladder, and aggravating the loss symptoms^(16)^.

Sun et al. showed in their work that pelvic floor muscle training is a procedure of choice for treating this complaint. They revealed that incontinent children had lower anal canal pressures at rest, and that after pelvic floor muscle training, there was no improvement in clinical outcomes^(7)^.

Combined therapy (enuresis alarm, bladder training, motivational therapy, and pelvic floor muscle training) is more effective than each component used alone or than pharmacotherapy^(17)^.

Children's involvement and participation are very important for successful results, since their participation occurs in a continuous way through a reeducation process and awareness of their problems^(8)^. Therefore, dialogue and patience were fundamental for parents' explanation on this voiding alteration. Further studies, with a greater number of patients, are necessary to prove the importance of pelvic floor training in children with UI.

The selection of nonmonosymptomatic children and the time consumed by pelvic floor muscle training are limitations of the study.

Behavioral modification and pelvic floor muscle training should be offered due to good results, absence of side effects, and good acceptance by the children. Future studies should be done to confirm if this treatment would be a first-line of therapy.

## CONCLUSION

In this study, the pelvic floor muscle training treatment was shown to be effective, noninvasive, and easily accepted by children. Behavioral therapy and pelvic floor training showed significantly higher improvement of urinary incontinence when compared to the use of oxybutynin and behavioral therapy of nonmonosymptomatic enuresis.
